# Evaluation of Health Risks Attributed to Toxic Trace Elements and Selenium in Farmed Mediterranean Mussels from Türkiye and Bulgaria

**DOI:** 10.1007/s12011-024-04084-w

**Published:** 2024-02-01

**Authors:** Hande Dogruyol, Suhendan Mol, Şafak Ulusoy, Alexander Atanasoff

**Affiliations:** 1 Department of Food Safety, Faculty of Aquatic Sciences, Istanbul University, Istanbul, 34134 Türkiye; 2 Department of Seafood Processing Technology, Faculty of Aquatic Sciences, Istanbul University, Istanbul, 34134 Türkiye; 3 Department of Animal Husbandry, Faculty of Veterinary Medicine, Trakia University, Stara Zagora, 6000 Bulgaria

**Keywords:** Mussel, Health risk, Toxic metal, Selenium, MOE, Food safety

## Abstract

Farmed mussels accumulate contaminants from their production environment rather than releasing them into water. This study reveals potential health risks associated with selenium, cadmium, mercury, and lead resulting from the consumption of mussels (*Mytilus galloprovincialis*) cultured along the coasts of Türkiye and Bulgaria. The concentrations of Se and toxic trace metals were measured by inductively coupled plasma mass spectrometry (ICP-MS). The detection limits (LOD) were 0.100, 0.015, 0.025, and 0.180 µg/kg for Se, Cd, Hg, and Pb, respectively. The mean Se concentrations were between 1.305 and 1.957 µg/g, and toxic metals were below the maximum limits. Due to Turkish and Bulgarian consumers’ limited mollusk consumption, mussels could only provide a maximum of 7.35% of the daily Se need. THQ and TTHQ of Se, Cd, and methyl-Hg were below 1, indicating that farmed mussels were safe for consumption. Percent PTWI values were calculated only for Cd and MeHg, as the PTWI value for Pb was discarded by the authorities and not determined for Se. Accordingly, weekly mussel consumption did not pose any risks. The margin of exposure approach was used to evaluate Pb intake. MOE-SBP and MOE-NE were significantly higher than 10, designating no significant health risks. Long-term consumption of mussels also does not pose a carcinogenic risk regarding the TR index calculated between 10^−5^ and 10^−6^ for Pb. Positive HBV_Se_ (10.13–37.27) indicated that Se in mussels overcame Hg-related potential health concerns. Consequently, mussels grown in Türkiye and Bulgaria did not pose a risk for human consumption, based on current risk analysis methods.

## Introduction

Mediterranean mussels are known to be highly nutritious and beneficial to health. Similar to other bivalve mollusks, they are high in protein and low in fat, carbohydrate, and cholesterol. Omega-3 polyunsaturated fatty acids are the predominant fatty acids in mussels, and they contain nutritionally important minerals besides being rich in astaxanthin and beta-carotenes [1, 2]. On the other hand, they feed by filtering substances from the water and accumulate materials from the aquatic environment in their tissues. Therefore, mussels living in polluted waters may cause bacterial and viral infections, as well as the transmission of biotoxins, industrial pollutants, and toxic metals to humans through the food chain [3]. Although metals are naturally found in the environment, industrial and urban development and uncontrolled waste disposal have led to an increase in anthropogenic fluxes, resulting in pollution of the coastal marine environment [4]. These metals may accumulate in seafood tissues and can be transferred to humans through consumption. Bioaccumulation of toxic metals in mussels transferred via the food chain is of great concern. Subjected to severe contamination, mussels may present health risks for consumers who frequently consume seafood [5].

Trace elements such as cadmium (Cd), lead (Pb), and mercury (Hg) are toxic even at low levels and have no biological tasks in the organism. Chronic exposure to such toxic metals can disrupt vital organ functions and eventually lead to cancer [6]. On the other hand, the trace element selenium (Se) is essential for humans and is found in high amounts in fish and shellfish. A compelling connection exists between Se and methylmercury (MeHg), the most toxic form of Hg. This binding mechanism is irreversible and interacts by inhibiting Se-dependent enzymes and eliminating Se for further selenoprotein synthesis. As a result, the amount of Se utilized in biological functions in the organism decreases, and the normal concentrations required for brain tissues and the endocrine system cannot be maintained [7]. Nevertheless, it is stated that a Se-rich diet prevents MeHg toxicity, creates a protective effect [7], and provides Se-aided demethylation of MeHg [8]. Therefore, Se health benefit value (HBV_Se_) is recommended as a useful index for assessing dietary exposures and implementing food safety measures [9].

Mediterranean mussels are popular street foods both in Türkiye and Bulgaria, available in shops, markets, and restaurants [10, 11]. Although mussels are well-liked seafood in both countries, total mollusk consumption quantities are lower than in many countries [12]. In recent years, significant improvements have been observed in mussel farming in Türkiye and Bulgaria [13–15]. Even when obtained through aquaculture, mussels are generally considered risky seafood as they filter their nutrients from seawater and can accumulate toxic metals in their edible tissues. However, metal concentrations in these sentinel organisms can vary greatly depending on seasonal, regional, and environmental conditions, posing a significant risk to food safety.

In order to reveal the health risks associated with farmed mussel consumption, Mediterranean mussels collected from four different farms were investigated seasonally. Se, Cd, Pb, Hg, and Se concentrations of mussels were determined. Estimated weekly intake (EWI); percent provisional tolerable weekly intake (%PTWI) for Cd, MeHg, and Se; and margin of exposure (MOE) for Pb were determined. The target hazard quotient (THQ) for Cd, MeHg, and Se and the target cancer risk (TR) for Pb were calculated. Based on the molar concentrations of Se and MeHg, the HBV_Se_ was also provided. In this study, the potential health risks of trace elements due to consuming Mediterranean mussels cultured in Türkiye and Bulgaria were evaluated for consumers in these regions. Determining possible food safety risks for these two countries, where mussel farming has increased in recent years, is also important to reveal the reliability of grown mussels and the suitability of mussel growing conditions.

## Material and Methods

### Experimental Sampling

**Fig. 1:**
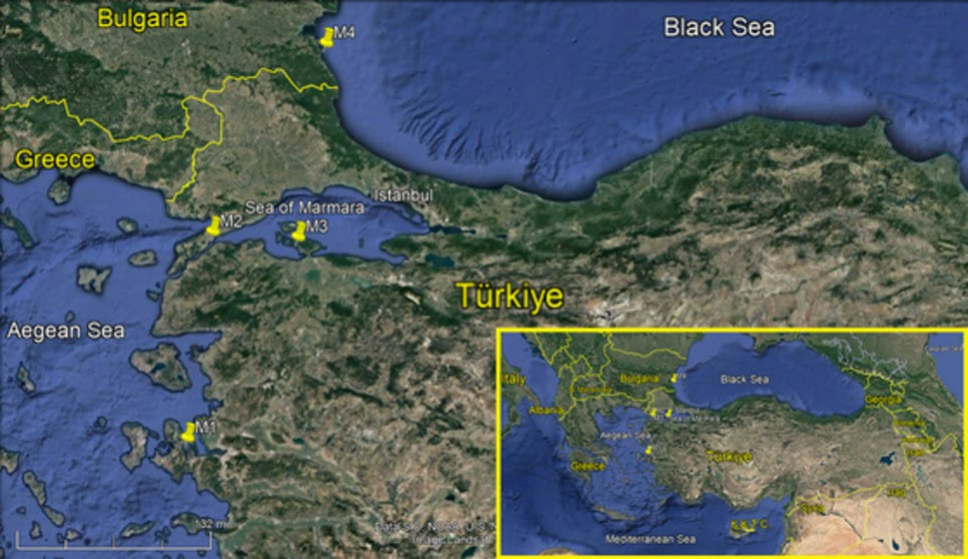
The locations of mussel farms as indicated M1, M2, M3, and M4

Cultured Mediterranean mussels (*Mytilus galloprovincialis*) were collected from four different farms. The farms coded M1, M2, and M3 are located on the coasts of Türkiye, and M4 is on the Bulgarian coast (Fig. 1). Sampling was carried out quarterly from summer to spring in 2019–2020. At least 100 individuals were used from each farm for each season. After the mussels were transferred to the laboratory, their average lengths and weights were measured (Table 1). Then, the shells of the mussels were removed, and the flesh was homogenized. Samples were frozen at − 24 °C in plastic jars until the analysis. Metal equipment was not used at any stage.

**Table 1:** The mean length (L) and weight (W) of the mussels from different farms (M1, M2, M3, and M4) according to seasons

	M1		M2		M3		M4	
	L (cm)	W (g)	L (cm)	W (g)	L (cm)	W (g)	L (cm)	W (g)
Summer	6.81 ± 0.56	17.60 ± 3.98	7.07 ± 0.36	22.70 ± 5.89	7.27 ± 0.37	21.30 ± 4.72	6.13 ± 0.65	14.97 ± 3.57
Fall	6.82 ± 0.58	18.60 ± 6.40	6.88 ± 0.63	17.10 ± 3.07	7.93 ± 0.37	23.60 ± 3.03	4.98 ± 0.72	8.30 ± 3.23
Winter	5.48 ± 0.68	11.30 ± 4.06	6.84 ± 0.45	22.60 ± 6.85	6.94 ± 0.41	20.90 ± 5.11	4.79 ± 0.50	8.30 ± 1.77
Spring	5.78 ± 0.37	9.00 ± 1.49	4.88 ± 0.50	9.40 ± 2.63	7.45 ± 0.47	23.8 ± 4.66	5.69 ± 0.27	10.11 ± 1.12

### Extraction and Instrumental Procedure

The analyses were carried out according to AOAC 999.10. From each mussel sample, approx. 0.5 g was weighed in a Teflon digestion vessel followed by the addition of 7 mL of 65% nitric acid (HNO_3_) and 1 mL of 30% hydrogen peroxide (H_2_O_2_). Then, ethos D (Plus 1) microwave lab station (Milestone Inc., Monroe, CT, USA) was used for digestion. The first and second steps of digestion were carried out at 0–200 °C for 20 min at 1000 W and 200–220 °C for 15 min at 1000 W, respectively. After waiting for 15 min at 220 °C at 1000 W, it was waited for 60 min to cool down. The volume of each dissolved sample was adjusted to 50 mL using deionized water. The concentrations of selenium (Se), cadmium (Cd), mercury (Hg), and lead (Pb) were measured by inductively coupled plasma mass spectrometry (ICP-MS; Perkin Elmer, NexION 1000, USA). The ICP-MS operating conditions were as indicated: nebulizer gas flow 0.92 L/min, radio frequency 1600 W, plasma gas 15.0 L/min, auxiliary gas 1.2 L/min. The instrument detection limits (LOD) were 0.1 µg/kg for Se, 0.015 µg/kg for Cd, 0.18 µg/kg for Pb, and 0.025 µg/kg for Hg. Catalogue numbers (part and lot numbers) of calibration standards which were obtained from Perkin Elmer Multi-element Standards (USA) were as follows: (1000 µg/mL in 2% HNO_3_) Se: PE: N9300182, Lot No: 23-115SEY1; (10 µg/mL in 5% HNO_3_) Cd and Pb: PE: N9300233, Lot No: CL4-108MKBY1; and (10 µg/mL in 5% HNO_3_) Hg: PE: N9300253, Lot No: CL10-37HGY1. High-purity deionized water was used for the preparation of standard solutions used for ICP-MS calibration. Validation of the method for accuracy was fulfilled with the certified reference material (Catalog no. ERM-CE278k, Geel, Belgium). As indicated in Table 2, the percent recovery values for standard reference material varied from 99 to 112%, demonstrating that the analytical processes used were accurate. The relative standard deviation (RSD) provides insight into whether the standard deviation is proportionally small or large in comparison to the mean of the dataset [16]. The RSD of measured concentration values (*n* = 3) had a high level of precision (RSD < 2.03%).

**Table 2:** Comparison between the certified and observed values (mg/kg) and the calculated recovery (%) of trace metal concentrations in standard reference material (*n* = 3)

	Certified values (mg/kg)	Observed values (mg/kg)		Recovery values (%)
		Mean ± SD (*n* = 3)	RSD (%)	
Se	1.620	1.840 ± 0.009	0.51	112.78
Cd	0.336	0.336 ± 0.010	0.30	99.70
Hg	0.071	0.074 ± 0.002	2.03	104.23
Pb	2.180	2.305 ± 0.004	0.18	105.73

### Health Risk Assessment

Annual mollusk consumption rates were taken into consideration since no particular consumption values were available for mussels. According to FAOSTAT 2020 data, the estimated food supply quantities of mollusks were 0.50 and 0.13 kg/capita per year in Türkiye and Bulgaria, respectively [12]. Thereby, the weekly ingestion rates (*IRw*) were determined as 9.61 g for Turkish and 2.50 g for Bulgarian consumers. EWI values for each metal were calculated by proportioning the metal concentration (*Cm*) to the *IRw* relative to the body weight (*BW*) (Eq. 1). The average body weight (*BW*) for adults was considered to be 70 kg, which is a “more realistic estimate” recommended by EFSA [17].

1 $$EWI=\frac{Cm*IRw}{BW}$$

The EWI values were compared with the provisional tolerable weekly intake (PTWI) of Cd and Hg. According to the conservative approach of the EFSA Scientific Panel on Pollutants in the Food Chain (CONTAM Panel), 80% of the total Hg in shellfish was assumed to be in the form of methylmercury (MeHg), and the PTWI for MeHg was set as 1.3 µg/kg *BW* [18]. For Cd, it was determined as 2.5 µg/kg *BW* [19]. Percent PTWIs were estimated accordingly.

With respect to Se and Pb, JECFA has not determined a certain PTWI value. In this study, dietary intake of selenium from cultured mussels was compared with the recommended Se intake value [20], which is 55 µg/day [21]. In the case of Pb, JECFA withdrew the established PTWI value of 25 µg/kg *BW* and considered it to no longer be health protective, because this most commonly used PTWI for Pb was associated with a decrease of at least 3 IQ points in children and a raise in systolic blood pressure of ~ 3 mmHg (0.4 kPa) in adults [22]. Therefore, the margin of exposure (MOE) method was used to determine the health risks associated with dietary Pb intake [23]. The MOE was determined by taking the 95th percentile lower confidence limit of the benchmark dose (BMDL) as the reference point and dividing it by the estimated exposure level of a population [24]. For the determination of MOE, the following equation was used [25]:

2 $${\text{MOE}}=\frac{BMDL}{EDI}$$

BMDL of an extra risk of 1% (BMDL_01_) for cardiovascular effect on systolic blood pressure (SBP) was set at 1.5 µg/kg BW per day, and the BMDL for an extra risk of 10% (BMDL_10_) for the nephrotoxicity (NE) was set at 0.63 µg/kg *BW* per day for adults [24]. Thus, MOE-SBP and MOE-NE were calculated separately for Pb. In order to determine the estimated daily intake (EDI), *IRw* was replaced by daily mollusk consumption (daily ingestion rate, *IRd*) in the EWI formula (Eq. 1).

Non-carcinogen and carcinogen health risks from the mollusk consumption were calculated using the target hazard quotient (THQ) and target cancer risk (TR) equations (Eqs. 3 and 4) given by [26]

3 $${\text{THQ}}=\frac{EF*ED*IFR*Cm}{BW*AT*RfD}*0.001$$

4 $${\text{TR}}=\frac{EF*ED*IFR*Cm*CSF}{BW*AT}*0.001$$

Exposure frequency (*EF*) and exposure duration (*ED*) were 350 days/year and 26 years, respectively. The following variables were used in the equations: *IFR* for food ingestion rate (g/day), *Cm* for metal concentration (mg/kg), *BW* for body weight (70 kg), *AT* for average exposure time (365 days/year × *ED* for non-carcinogens, and 365 days/year × 70 years for carcinogens). Additionally, *RfD* represents the oral reference dose (mg/kg per day), and *CSF* stands for the carcinogenic slope factor. The *RfD* was selected as 5 × 10^−3^ mg/kg per day for Se and 1 × 10^−4^ mg/kg per day for both Cd and MeHg [26]. The *CSF* was 8.5 × 10^−3^ (mg/kg per day)^−1^ for Pb [27]. THQ values were calculated for Se, Cd, and MeHg, whereas the TR index was determined for Pb. Since the adverse health effects like the changes in blood enzymes and/or in aspects of children’s neurological development due to exposure to inorganic Pb may occur at blood lead levels as low as to lack a discernible threshold, the US EPA [28] concluded that it is not appropriate to set an RfD for Pb. Therefore, THQ was not calculated for Pb.

Molar concentrations of Se and Hg (µmol/kg) were calculated by using their mass concentrations (mg/kg). Selenium health benefit value (HBV_Se_) was calculated as follows [9]:

5 $$\mathrm{HBV Se}=\frac{Se-Hg}{Se}*\left({\text{Se}}+{\text{Hg}}\right)$$

### Data Analyses

Statistical analyses were carried out by using SPSS v21.0 software (IBM SPSS Inc., Chicago, IL, USA). Samples were run in triplicates. Trace metal concentrations of each mussel sample were subjected to one-way ANOVA, and the differences were identified by using Tukey’s post hoc test. Mean values and standard deviations were calculated. The significance level was set at 0.05.

## Results and Discussion

### Trace Metal Concentrations

The concentrations of trace metals in mussels, collected from four different farms across various seasons, are presented in Table 3. The average Se concentrations of mussels cultured in different locations were between 1.305 and 1.957 µg/g. This element was found to be above 2 µg/g in mussels from farms in Turkish waters, especially in the spring. The Se concentrations in Turkish seawaters varied between 0.004 and 2.54 µg/L [29]. Se is utilized as a fertilizer component for growing wheat, and it is hard to control its spread in the environment [30]. Tokatlı et al. [31] reported that the high selenium concentrations, even in waters free from industrial pollution, indicated that the major source of selenium accumulation may be associated to sewage disposal and agricultural activities. Moreover, the Se content of foods varies substantially by geography [30]. Selenium concentrations in bivalve mollusks from various regions were reported to be 0.53 µg/g in *Mytilus edulis* from France [32], 0.284 µg/g in brown mussels (*Perna perna*) from Brazil [33], and 3.34 µg/g and 2.79 µg/g in *Saccostrea palmula* and *Crassostrea corteziensis* oysters from California [34], respectively. Differences in trace element levels among seafood species, sampled from diverse habitats, can be attributed to the influential factors of the food chain and biomagnification [35].

**Table 3:** Seasonal trace metals concentrations of mussels obtained from four different farms seasonally

Selenium (µg/g)				
	M1	M2	M3	M4
Summer	1.719^a,W^ ± 0.028	1.085^a,X^ ± 0.049	0.843^a,Y^ ± 0.010	0.800^a,Y^ ± 0.018
Fall	1.093^b,W^ ± 0.032	1.781^b,X^ ± 0.041	1.474^b,Y^ ± 0.010	1.311^b,Z^ ± 0.022
Winter	2.070^c,W^ ± 0.059	1.558^c,X^ ± 0.078	1.793^c,Y^ ± 0.021	1.792^c,Y^ ± 0.050
Spring	2.946^d,W^ ± 0.039	2.790^d,X^ ± 0.047	2.033^d,Y^ ± 0.053	1.317^b,Z^ ± 0.052
Annual	1.957 ± 0.039	1.804 ± 0.054	1.536 ± 0.024	1.305 ± 0.036
Cadmium (µg/g)				
	M1	M2	M3	M4
Summer	0.063^a,W^ ± 0.000	0.085^a,X^ ± 0.001	0.195^a,Y^ ± 0.004	0.244^a,Z^ ± 0.002
Fall	0.082^b,W^ ± 0.001	0.180^b,X^ ± 0.003	0.205^b,Y^ ± 0.002	0.429^b,Z^ ± 0.002
Winter	0.121^c,W^ ± 0.002	0.180^b,X^ ± 0.002	0.258^c,Y^ ± 0.002	0.244^a,Z^ ± 0.002
Spring	0.195^d,W^ ± 0.003	0.207^c,X^ ± 0.002	0.181^d,Y^ ± 0.002	0.376^c,Z^ ± 0.001
Annual	0.115 ± 0.002	0.163 ± 0.002	0.210 ± 0.002	0.323 ± 0.002
Mercury (µg/g)				
	M1	M2	M3	M4
Summer	0.085^a,W^ ± 0.004	0.031^a,XY^ ± 0.001	0.033^a,X^ ± 0.001	0.026^a,Y^ ± 0.001
Fall	0.034^b,W^ ± 0.001	0.025^b,X^ ± 0.001	0.020^b,Y^ ± 0.001	0.013^b,Z^ ± 0.001
Winter	0.032^b,W^ ± 0.001	0.017^c,X^ ± 0.001	0.019^bc,X^ ± 0.001	0.025^a,Y^ ± 0.002
Spring	0.315^c,W^ ± 0.004	0.032^a,X^ ± 0.001	0.017^c,Y^ ± 0.001	0.011^b,Z^ ± 0.001
Annual	0.117 ± 0.003	0.026 ± 0.001	0.022 ± 0.001	0.019 ± 0.001
Lead (µg/g)				
	M1	M2	M3	M4
Summer	0.091^a,W^ ± 0.007	0.091^a,W^ ± 0.001	0.036^a,X^ ± 0.002	0.058^a,Y^ ± 0.002
Fall	0.079^a,W^ ± 0.006	0.147^b,X^ ± 0.006	0.045^b,Y^ ± 0.002	0.091^b,Z^ ± 0.001
Winter	0.196^b,W^ ± 0.001	0.253^c,X^ ± 0.001	0.152^c,Y^ ± 0.003	0.152^c,Y^ ± 0.001
Spring	0.300^c,W^ ± 0.002	0.372^d,X^ ± 0.005	0.152^c,Y^ ± 0.001	0.183^d,Z^ ± 0.003
Annual	0.167 ± 0.004	0.216 ± 0.003	0.096 ± 0.002	0.121 ± 0.002

M1, M2, and M3: Farms in Türkiye. M4: Farm in Bulgaria

^a,b,c,d^Different lower case letters in the same column for each metal indicate a significant difference (*p* < 0.05) among the mean values of metal concentration for mussels from the same farm in different seasons

^W,X,Y,Z^Different capital letters in the same row indicate a significant difference (*p* < 0.05) among the mean values of metal concentration for mussels from different farms in the same season

The average Cd levels were in the range of 0.115–0.323 µg/g, staying under the threshold of 2 µg/g [36], and all samples from various locations in different seasons contained Cd below this limit. Belivermiş et al. [37] studied the metal concentrations of *M. galloprovincialis* from Turkish coasts and reported the Cd concentration between 0.09 and 3.32 µg/g. The European Commission [38] has set the maximum Hg limit for crustaceans, mollusks, and muscle meat of some fish at 0.50 mg/kg wet weight. In this study, the mean Hg concentrations ranged between 0.019 and 0.117 µg/g (Table 3), well below this limit. Mean Pb concentrations (0.096–0.216 µg/g) were also below the CAC [36] recommended limit of 0.30 µg/g, and only the spring M2 sample (0.372 µg/g) was slightly above the limit. Stancheva et al. [39] reported Cd, Hg, and Pb concentrations of *M. galloprovincialis* from a shellfish farm in Kranevo, Bulgaria, as 0.09, 0.32, and 0.18 µg/g, respectively. In another study conducted in Bulgaria, the average Cd concentration in mussels (*M. galloprovincialis*) from the Gulf of Varna was reported to be 0.280 µg/g, while Hg and Pb concentrations were 0.017 µg/g and 0.251 µg/g, which were similar to those reported in our study [40]. Kucuksezgin et al. [41] monitored the heavy metal concentrations of transplanted mussels (*M. galloprovincialis*) on the Aegean coast of Türkiye. They reported similar Cd (0.46–3.35 µg/g) and Hg (0.052–0.192 µg/g) concentrations, but higher Pb levels (1.06–4.43 µg/g) than our findings. Kuplulu et al. [42] investigated metal contaminations in seafood from Turkish coasts. They reported the mean Cd, Hg, and Pb concentrations (μg/g) of *M. galloprovincialis* as 0.143, 0.079, and 0.405 in Aegean coast samples and 0.112, 0.203, and 0.366 in Mediterranean coast samples, respectively. The average Cd and Pb concentrations in wild mussels from the Aegean coast of Türkiye were reported to be 0.25 and 0.98 μg/g, respectively [43]. Previously reported Pb concentrations in Mediterranean mussels were higher than in this study and above the recommended limit of 0.30 µg/g [42, 44]. Similarly, the higher annual Pb concentrations (0.2977–0.7743 µg/g) were reported in *M. galloprovincialis* farmed in Cala Iris, and the significant effect of seasonal variations on metal concentrations was mentioned [40]. In the present study, it was also determined that the mean values of toxic metal concentrations detected in different seasons were significantly (*p* < 0.05) different from each other (Table 3).

A significant portion of the mussels consumed in Türkiye are collected from the Marmara Sea, and it was reported that Pb concentrations in these mussels were above the limits [45, 46]. Acarlı et al. [47] sampled mussels from the Yalova coast of the Marmara Sea and reported that the Yalova region is not safe for both juvenile collection and mussel farming. The fact that Pb concentrations can reach high values shows the importance of the correct selection of mussel-growing areas. The quality of the mussel products mainly depends on the water quality. In addition to food availability, factors such as breeding season and biochemical elements are also crucial parameters to consider [1]. Kacar et al. [48] used mussels as a pollution indicator and collected mussels from densely populated, industrial, commercial, and maritime transportation and industrial discharge sites on the Turkish coast. They stated that the fact that Pb concentrations can reach high values showed the importance of choosing the right areas where mussels were cultured. In this study, the trace element levels were found to be at levels suitable for human consumption in the samples taken from mussel farms on the Aegean coast of Türkiye and the Sozopol coast of Bulgaria.

Monitoring and health risk assessments are essential to ensure the safety of farmed mussel products. Moreover, it is important to provide practical implications for the aquaculture industry by addressing potential mitigation strategies to minimize toxic metal accumulation in mussel aquaculture. In the comprehensive framework for controlling contaminants in mussels cultivated for human consumption, measures such as monitoring and managing water quality [49], establishing surveillance plans, identifying and controlling pollution sources [50], conducting soil analyses near the cultivation areas [51], implementing accepted methodologies such as “Life Cycle Assessment” [52], and dissipating metal accumulation through the selection of different farming locations [53] should be included.

Annual fluctuations in trace element concentrations serve as evidence that variations are influenced by natural geographical characteristics and anthropogenic activities. However, it is quite difficult to make a definite distinction between these factors unless there is obvious evidence. In this study, four different farms located in three different seas were investigated (Fig. 1). M1 is situated on the borders of one of the highly urbanized cities in the Aegean Sea. M2 and M3 are located in the Sea of Marmara, which is an inland sea and a link between the Mediterranean and Black Sea semi-enclosed basins [54]. M4 is located in the west end of Black Sea remaining in Bulgarian coasts. The lowest Se and Hg levels were observed in the mussels obtained from M4, while the highest concentrations were found in M1. Conversely, M4 had the highest Cd levels, whereas M1 had the lowest. Additionally, M2 had the highest Pb levels, while M3 had the lowest (Table 3).

### Health Risk Estimation

Seafood consumption varies significantly among different populations influenced by their eating habits. Therefore, although limit values have been established for toxic element concentrations in fish and other seafood, it is important to consider the amount of ingestion to estimate potential health risks associated with toxic elements [55, 56].

Selenium is an element found in seafood, and excessive dietary Se intake may be harmful to health [57]. However, selenium-related health problems are mostly associated with its deficiency, and insufficient selenium intake is reported to be one of the main factors in many health problems [58]. Thus, the daily dietary intake of Se is set at 55 µg, with a recommendation of no more than 400 µg/day to avoid long-term side effects [21]. In this study, dietary intake of Se from cultured mussels was compared with the recommended Se intake value of 55 µg/day to meet the nutrient needs of a healthy person. Accordingly, the maximum contribution of mussels in meeting the daily Se requirement was found to be 7.35% due to the low consumption of mollusks in both countries (Table 4). Since Se is a trace element with the potential to prevent diseases in humans, dietary intake at low to moderate levels is essential to support life. Therefore, JECFA has not set a specific PTWI for dietary selenium intake, and hence, there is no percent PTWI calculation in Table 4.

**Table 4:** Estimated daily intake (EDI), daily intake coverage percentage, and target hazard quotient (THQ) calculations of Se determined in cultivated mussels in different farms (M1, M2, M3, and M4) seasonally

Selenium (Se)				
		EDI (µg/kg)	% daily intake*	THQ
M1	Summer	0.034	4.29	0.006
	Fall	0.021	2.73	0.004
	Winter	0.041	5.17	0.008
	Spring	0.058	7.35	0.011
M2	Summer	0.021	2.71	0.004
	Fall	0.035	4.45	0.007
	Winter	0.031	3.89	0.006
	Spring	0.055	6.96	0.010
M3	Summer	0.017	2.10	0.003
	Fall	0.029	3.68	0.006
	Winter	0.035	4.48	0.007
	Spring	0.040	5.07	0.008
M4	Summer	0.004	0.52	0.001
	Fall	0.007	0.85	0.001
	Winter	0.009	1.16	0.002
	Spring	0.007	0.86	0.001

^*^For the determination of daily Se intake fulfilment, recommended dietary allowance (RDA) of 55 µg/day [21] was taken into consideration

It was reported that 80% of the total Hg in shellfish is in the form of MeHg, the most toxic form of mercury [18]. Accordingly, MeHg was calculated as 80% of Hg concentrations and used in the EWI calculations. In this study, the EWIs of Cd and MeHg were compared with the PTWIs given by EFSA [18, 19]. The percent PTWI values of Cd (Table 5) and MeHg (Table 6) were found below 100%, showing no potential risks. Similarly, the estimated daily and weekly intakes of Cd and Hg in mussels from the Black Sea coast of Türkiye were reported to be below the permissible intakes [59]. Chiesa et al. [60] collected mussels from the Italian market and similarly reported that there was no significant risk to the average Italian consumer due to their metal content. Omeragic et al. [61] reported that the average weekly heavy metal exposure via date mussels (*Lithophaga lithophaga*) was lower than the provisional tolerable weekly intake due to low consumption of mollusks in Bosnia and Herzegovina. They emphasized the need for studies on the frequency of seafood consumption to make a reliable risk assessment. Indeed, Belivermiş et al. [37] reported that mussel consumption in Türkiye is low but can be quite high in coastal areas. Therefore, the potential risk may be higher for consumers in these regions. The very low percent PTWIs calculated in this study suggest that farmed mussels would be generally safe for consumption.

**Table 5:** Estimated daily and weekly intakes (EDI and EWI), percent provisional tolerable weekly intake (%PTWI), and target hazard quotient (THQ) calculations of Cd determined in cultivated mussels in different farms (M1, M2, M3, and M4) seasonally

Cadmium (Cd)					
		EDI (µg/kg)	EWI (µg/kg)	%PTWI*	THQ
M1	Summer	0.001	0.009	0.35	0.012
	Fall	0.002	0.011	0.45	0.015
	Winter	0.002	0.017	0.66	0.023
	Spring	0.004	0.027	1.07	0.037
M2	Summer	0.002	0.012	0.47	0.016
	Fall	0.004	0.025	0.99	0.034
	Winter	0.004	0.025	0.99	0.034
	Spring	0.004	0.028	1.14	0.039
M3	Summer	0.004	0.027	1.07	0.037
	Fall	0.004	0.028	1.13	0.038
	Winter	0.005	0.035	1.42	0.048
	Spring	0.004	0.025	0.99	0.034
M4	Summer	0.001	0.009	0.35	0.012
	Fall	0.002	0.015	0.61	0.021
	Winter	0.001	0.009	0.35	0.012
	Spring	0.002	0.013	0.54	0.019

^*^Provisional tolerable weekly intake (PTWI) of 2.50 µg/kg *BW* for Cd [19] was used for the calculations

**Table 6:** Estimated daily and weekly intakes (EDI and EWI), percent provisional tolerable weekly intake (%PTWI), and target hazard quotient (THQ) calculations of MeHg determined in cultivated mussels in different farms (M1, M2, M3, and M4) seasonally

Methylmercury (MeHg)					
		EDI (µg/kg)	EWI (µg/kg)	%PTWI*	THQ
M1	Summer	0.0013	0.0093	0.72	0.013
	Fall	0.0005	0.0037	0.29	0.005
	Winter	0.0005	0.0035	0.27	0.005
	Spring	0.0049	0.0346	2.66	0.047
M2	Summer	0.0005	0.0034	0.26	0.005
	Fall	0.0004	0.0027	0.21	0.004
	Winter	0.0003	0.0019	0.14	0.003
	Spring	0.0005	0.0035	0.27	0.005
M3	Summer	0.0005	0.0036	0.28	0.005
	Fall	0.0003	0.0022	0.17	0.003
	Winter	0.0003	0.0021	0.16	0.003
	Spring	0.0003	0.0019	0.14	0.003
M4	Summer	0.0001	0.0007	0.06	0.001
	Fall	0.0001	0.0004	0.03	0.001
	Winter	0.0001	0.0007	0.05	0.001
	Spring	0.0000	0.0003	0.02	0.000

^*^Provisional tolerable weekly intake (PTWI) of 1.30 µg/kg *BW* for MeHg [18] was used for the calculations

**Fig. 2:**
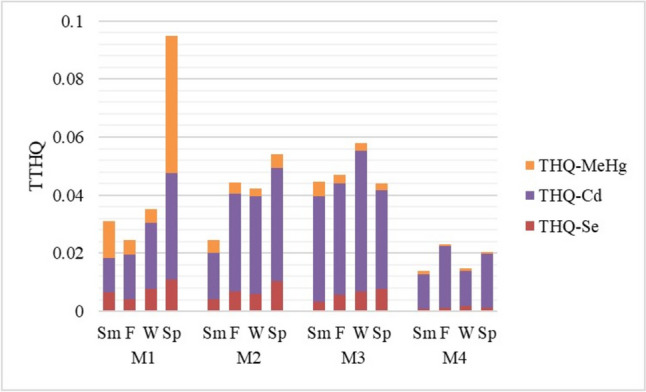
Total target hazard quotient (TTHQ) of MeHg, Cd, and Se in mussels obtained from different farms across seasons (Sm, summer; F, fall; W, winter; Sp, spring)

Another definition used to determine possible health risks due to metal exposure is THQ, and a value above 1 indicates a potential concern [27]. In this study, it was observed that all THQ values in farmed mussels in different locations were well below 1 (Table 4, 5, and 6). It was reported that the THQ values of farmed [62] and wild [63] mussels in Bulgaria were below 1, and there was no potential health risk associated with mussel consumption for the Bulgarian consumers. Similarly, Varna Bay mussels were reported to be safe for consumption due to their low THQ values such as 0.0001 for Cd and 0.0006 for Hg, well below 1 [64]. In the present study, even the sum of the THQ values (TTHQ) of Se, Cd, and MeHg for farmed mussels at each location was below 1 (Fig. 2). Therefore, no potential health risk from the consumption of farmed mussels has been foreseen with the current consumption levels in Türkiye and Bulgaria.

With regard to Pb, percent PTWI and THQ were not calculated because the PTWI and RfD are no longer in use. The former PTWI value of 25 µg/kg *BW* used to determine the potential health risks associated with dietary lead intake was withdrawn on the grounds that it could lead to lower intelligence scores in children and increased systolic blood pressure in adults [24]. EFSA hereby deems the utilization of MOE appropriate for evaluating the risks associated with Pb in food [24]. The MOE approach might be used to assess the level of health risk of a carcinogenic and genotoxic contaminant, and it is the ratio of the benchmark dose’s lower confidence limit (BMDL) and estimated human consumption of a substance. In fact, MOE indicates the level of health concern without quantifying the risk and casts light on the ranking of toxic substances [65]. In the case of Pb, the BMDLs were based on dose–response assessments in humans. The CONTAM panel inferred that a MOE value of 10 or higher ensures no clinically significant risks on systolic blood pressure (SBP) or chronic kidney diseases/nephrotoxicity (NE). Also, MOE values below 1 were reported to indicate very low risk and no concern from a public health perspective [23]. In summary, the larger the MOE, the lower the concern for human health. EFSA [24] also suggested BMDL of an extra risk of 1% (BMDL_01_) for potential cardiovascular effects (SBP) as 1.50 µg/kg BW and an extra risk of 10% (BMDL_10_) for potential nephrotoxicity effects (NE) as 0.63 µg/kg BW. In this study, the MOE-SBP (BMDL_01_) and MOE-NE (BMDL_10_) values were higher than 10, indicating no significant health risks for humans (Table 7). In a similar study, the MOE values of mussels sold in Thailand were above 1, showing very low risks [66]. Okoye et al. [67] examined the effects of heavy metals in fish and meat on human health. Taking into account the Pb-MOE values, they reported that the Pb level in these foods would not cause a negative effect on the kidneys. The health risk associated with the consumption of vegetables and grains was evaluated by calculating MOE, and it was reported that the health risk due to Pb was insignificant since the MOEs were above 1 [68]. In China, the MOE was used to evaluate the health risks of residents in mining areas due to dietary Pb intake. The MOE of red meat was reported to be very low, indicating a high health risk due to Pb contamination, and the consumers were encouraged to consume more fish [69]. Even though the MOE is not an exact proof of health risk, lower MOE values indicate a possible concern, and studies on various foods are needed [65].

**Table 7:** Estimated daily intake (EDI), margin of exposure on systolic blood pressure (MOE-SBP), margin of exposure on nephrotoxicity (MOE-NE), and target carcinogenic risk calculations of Pb determined in cultivated mussels in different farms (M1, M2, M3, and M4) seasonally

Lead (Pb)					
		EDI (µg/kg)	MOE-SBP*	MOE-NE**	TR
M1	Summer	0.002	840.47	353.00	5.39E − 06
	Fall	0.002	968.14	406.62	4.68E − 06
	Winter	0.004	390.22	163.89	1.16E − 05
	Spring	0.006	254.94	107.08	1.78E − 05
M2	Summer	0.002	840.47	353.00	5.39E − 06
	Fall	0.003	520.29	218.52	8.71E − 06
	Winter	0.005	302.30	126.97	1.50E − 05
	Spring	0.007	205.60	86.35	2.20E − 05
M3	Summer	0.001	2124.52	892.30	2.13E − 06
	Fall	0.001	1699.62	713.84	2.67E − 06
	Winter	0.003	503.18	211.33	9.01E − 06
	Spring	0.003	503.18	211.33	9.01E − 06
M4	Summer	0.000	5068.97	2128.97	3.44E − 06
	Fall	0.000	3230.77	1356.92	5.39E − 06
	Winter	0.001	1934.21	812.37	9.01E − 06
	Spring	0.001	1606.56	674.75	1.08E − 05

^*^MOE-SBP: Margin of exposure calculated by using BMDL of an extra risk of 1% (BMDL_01_) for adverse effect on systolic blood pressure (SBP) which is 1.5 µg/kg *BW* per day for adults[24]

^**^MOE-NE: Margin of exposure calculated by using BMDL for an extra risk of 10% (BMDL_10_) for the nephrotoxicity (NE) which is 0.63 µg/kg *BW* per day for adults[24]

In this study, the target carcinogenic risk (TR) for potential hazards from Pb intake through consumption of farmed mussels was also calculated. TR index is defined as the increased likelihood of developing cancer during an individual’s lifetime due to exposure to a potential carcinogen [70]. For carcinogenic toxic metals, such as Pb, TR values above 10^−4^ are considered to pose high cancer risk and unacceptable, while values below 10^−6^ are regarded as negligible. TR values within the range of 10^−4^ to 10^−6^ are considered acceptable risk [71]. In the present study, all TR values for Pb were between 10^−5^ and 10^−6^, indicating that farmed mussel samples were acceptable for human consumption (Table 7). Accordingly, Peycheva et al. [63] reported the TR indexes for Pb to be below 10^−6^ and concluded that mussels from the Black Sea coast of Bulgaria did not pose a carcinogenic risk. Similarly, the TR value for Pb was determined as 10^−9^ in farmed *M. galloprovincialis* from Bulgaria, which did not pose a health risk to humans in terms of Pb concentrations [72].

### Selenium Health Benefit Value (HBV_Se_)

**Fig. 3:**
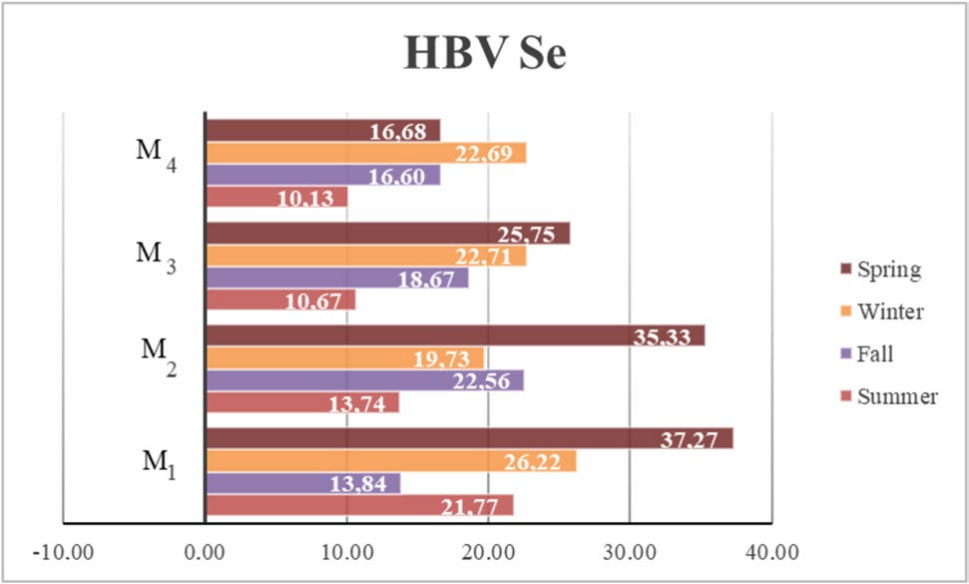
Comparison of HBV_Se_ indexes of the mussel farms (M1, M2, M3, and M4) according to different seasons

Selenium can reduce oxidative stress induced by mercury, compete with it, and suppress its absorption [9, 73]. Also, directing mercury to less sensitive organs and converting mercury ions to less harmful forms are the other benefits of Se element [73]. In the context of the Se–Hg relationship, various indexes have been utilized, including the Se:Hg molar ratio and benefit-risk value. However, a relatively new risk assessment criterion, HBV_Se_, may provide clearer results as it allows for a more accurate calculation of health risk in products containing high concentrations of Hg and Se [74]. This value includes the benefits of Se as well as its protective effect against Hg, thus helping to distinguish between seafood that should be limited during pregnancy and breastfeeding due to potential risks and those that should be eaten for the neurodevelopment of the child. Thus, helps to prevent avoiding foods that are essential for good health because of concerns about potential risks [75]. Consumption of HBV_Se_-positive seafood provides protection against methylmercury-related risks. On the other hand, regular consumption of seafood with a negative HBV_Se_ value may lead to methylmercury-related health problems, especially for mothers with low selenium intake, and HBV_Se_ is used as the indicator of excess amount of Se over MeHg [9]. Selenium deficiency has been reported to have an effect on MeHg-induced toxicity [76]. HBV_Se_ values of all samples analyzed during the whole study were positive and ranged between 10.13 and 37.27 (Fig. 3), indicating that the Se content of mussels overcomes Hg-related health concerns. Similarly, Sepúlveda et al. [34] reported HBV_Se_ for oysters *S. palmula* and *C. corteziensis* in the range of 19.23–42.28 and 17.82–35.30, respectively, and reported that Se concentrations provided a benefit over Hg. They also mentioned that high Se levels can reduce the risk of possible toxicity caused by Hg and that these seafood products should be considered safe for the human diet. Sabino et al. [77] reported that tropical fish in Seychelles were not risky despite their high Hg content considering their HBV_Se_ value. Similarly, the possibility of MeHg toxicity was reported to be negligible, due to the positive HBV_Se_ values of fish from the coast of Long Island, New York [78]. In various Atlantic marine fish species, HBV_Se_ values were reported between 2.1 and 7.1 [79], and these values were lower than those found for farmed mussels in our study. Meanwhile, it should not be ignored that each MeHg molecule may not bond with a Se molecule. Prior to Se intervention, it is highly probable that Hg molecules will come into contact with sulfur, potentially leading to toxicity [7]. However, due to the high HBV_Se_ values detected in our study, it was concluded that Hg in cultured mussels does not pose a significant risk to health. Recent studies [74, 80] have emphasized that worldwide laboratory studies will be needed for criteria such as HBV_Se_ to be well recognized and that more studies should be conducted using the HBV_Se_ value.

In Table 8, the previous studies examining trace elements in both wild and farmed mussels are presented. Selenium concentration was neglected in almost all of these studies, and HBV_Se_ was not calculated in any of them. Moreover, the MOE was not calculated and determined by using the BMDL of SBP or NE in studies originating from Türkiye and Bulgaria.

**Table 8:** Recent studies investigating trace element concentrations in mussel species from the coasts of Türkiye, Bulgaria, and other regions of the world

Region	Reference study	Wild/farmed	Trace element concentration	EDI/EWI/%PTWI	MOE (Pb)	THQ/TTHQ/HI	TR (Pb)	Se concentration	HBV_Se_
Turkish coasts	This study	Farmed*	+	+	+	+	+	+	+
	Acarli et al. [47]	Wild*	+	-	-	-	-	-	-
	Katalay et al. [81]	Wild*	+	-	-	-	-	-	-
	Kuplulu et al. [42]	Wild*	+	-	-	-	-	-	-
	Bat et al. [59]	Wild*	+	+	-	-	-	-	-
	Gedik [82]	Wild*	+	+	-	+	-	-	-
	Belivermis et al. [37]	Transplanted*	+	+	-	-	-	-	-
	Kacar et al. [48]	Wild*	+	+	-	-	-	-	-
	Kucuksezgin et al. [41]	Transplanted*	+	-	-	-	-	-	-
	Çulha et al. [45]	Wild*	+	-	-	-	-	-	-
	Mol and Üçok Alakavuk [46]	Wild*	+	-	-	-	-	-	-
	Sunlu [43]	Wild*	+	-	-	-	-	-	-
Bulgarian coasts	Peycheva et al. [63]	Wild*	+	+	-	+	+	-	-
	Peycheva et al. [72]	Farmed*	+	+	-	+	+	-	-
	Peycheva et al. [62]	Farmed and wild *	+	+	-	+	+	-	-
	Zhelyazkov et al. [64]	Wild*	+	+	-	+	-	-	-
	Stancheva et al. [15]	Farmed and wild*	+	-	-	-	-	-	-
Other locations	Tanaviyutpakdee and Karnpanit [66]	Wild *Perna viridis*	+	-	+	+	-	-	-
	de Oliveira et al. [33]	Wild *Perna perna*	+	+	-	-	-	-	-
	Ferraris et al. [32]	Farmed *Mytilus edulis*	+	+	-	-	-	+	-
	Omeragic et al. [61]	Wild *L. lithophaga*	+	+	+	+	-	-	-
	Azizi et al. [40]	Wild*	+	-	-	-	-	-	-
	Chiesa et al. [60]	Wild*	+	+	+	+	-	-	-
	Roméo et al. [83]	Wild *	+	-	-	-	-	-	-

^*^Mytilus galloprovincialis

## Conclusion

In recent years, Türkiye and Bulgaria have made significant progress in mussel cultivation within Eastern Europe. Due to mussels’ tendency to accumulate substances and trace elements from their environment, frequent consumption raises significant concerns regarding food safety. The metal concentrations of mussels did not exceed the limits established by the European regulation. According to the recent indexes and updated methods, mussels farmed on Turkish and Bulgarian coasts were found safe for human ingestion. No potential health risks were determined based on annual and seasonal evaluations. The TR values derived from Pb intake in mussels were determined to be acceptable, indicating no long-term cancer risk. Furthermore, it could be anticipated that the positive HBV_Se_ value obtained in this study might mitigate the risks typically associated with MeHg exposures in mussels. The present study is original as it provides new information on the potential health risks of farmed mussels.

## Data Availability

The datasets generated during and/or analysed during the current study are not publicly available due to security concerns regarding data misuse, but are available from the corresponding author on reasonable request.
